# Evaluation of long noncoding RNA MALAT1 as a candidate blood-based biomarker for the diagnosis of non-small cell lung cancer

**DOI:** 10.1186/1756-0500-6-518

**Published:** 2013-12-06

**Authors:** Daniel Gilbert Weber, Georg Johnen, Swaantje Casjens, Oleksandr Bryk, Beate Pesch, Karl-Heinz Jöckel, Jens Kollmeier, Thomas Brüning

**Affiliations:** 1 Institute for Prevention and Occupational Medicine of the German Social Accident Insurance, Institute of the Ruhr-Universität Bochum (IPA), Bürkle-de-la-Camp-Platz 1, 44789 Bochum, Germany; 2 Institute of Medical Informatics, Biometry and Epidemiology, University of Duisburg-Essen, Hufelandstrasse 55, 45122 Essen, Germany; 3 HELIOS Clinic Emil von Behring, Respiratory Diseases Clinic Heckeshorn, Walterhöferstrasse 11, 14165 Berlin, Germany

**Keywords:** Lung, Cancer, NSCLC, Biomarkers, lncRNA, *MALAT1*, Minimally-invasive, Sensitivity, Specificity

## Abstract

**Background:**

The long noncoding RNA *MALAT1* (metastasis-associated lung adenocarcinoma transcript 1) is described as a potential biomarker for NSCLC (non-small cell lung cancer). Diagnostic biomarkers need to be detectable in easily accessible body fluids, should be characterized by high specificity, sufficient sensitivity, and robustness against influencing factors. The aim of this study was to evaluate the performance of *MALAT1* as a blood based biomarker for NSCLC.

**Results:**

*MALAT1* was shown to be detectable in the cellular fraction of peripheral human blood, showing different expression levels between cancer patients and cancer-free controls. For the discrimination of NSCLC patients from cancer-free controls a sensitivity of 56% was calculated conditional on a high specificity of 96%. No impact of tumor stage, age, gender, and smoking status on *MALAT1* levels could be observed, but results based on small numbers.

**Conclusions:**

The results of this study indicate that *MALAT1* complies with key characteristics of diagnostic biomarkers, i.e., minimal invasiveness, high specificity, and robustness. Due to its relatively low sensitivity *MALAT1* might not be feasible as a single biomarker for the diagnosis of NSCLC in the cellular fraction of blood. Alternatively, *MALAT1* might be applicable as a complementary biomarker within a panel in order to improve the entire diagnostic performance.

## Background

Lung cancer is the leading cause of cancer death worldwide [1] with NSCLC (non-small cell lung cancer) as the most prominent subgroup accounting for approximately 80% of all lung cancer cases. Commonly, the disease is detected in late stages resulting in short survival rates, whereas for patients with early-stage lung cancer longer survival rates could be observed [2]. Thus, the detection of lung cancer in early stages when clinical symptoms have not yet occurred appears to be a promising opportunity to decrease mortality, because in more cases a curative therapy might become possible.

In principal, biomarkers should be feasible for the detection of cancer in early stages. Thus, a major aim in cancer research is the identification of proper biomarkers. Key characteristics of diagnostic biomarkers among others are: (i) minimally-invasive to measure the biomarker in easily accessible body fluids, (ii) high specificity to avoid false-positive results in cancer-free individuals, (iii) sufficient sensitivity to detect the tumors, and (iv) robustness against potential influencing factors.

In recent years biomarker research focused on noncoding RNAs (ncRNAs), in particular microRNAs (miRNAs). MiRNAs are small RNA molecules with a length of ~ 22 nucleotides (nt), playing a central role in the regulation of gene expression [3] and acting as tumor suppressors or oncogenes in cancer [4]. Several studies show the feasibility of using miRNAs as biomarkers in body fluids for the diagnosis of lung cancer [5-8]. However, there is a lack of consistent results between studies focused on the identification of miRNAs as biomarkers [9]. Thus, the discovery of alternative or complementing biomarkers is essential.

In addition to miRNAs, long noncoding RNAs (lncRNAs) are a promising alternative within the group of ncRNAs. LncRNAs are commonly described as RNA molecules with a length > 200 nt, playing regulatory and structural roles in biological processes. As lncRNAs are implicated as tumor suppressors and oncogenes [10], they might be feasible as diagnostic biomarkers [11]. Currently, only few lncRNAs have been described as candidate biomarkers in human body fluids [10]. *HULC* (highly up-regulated in liver cancer) is highly expressed in hepatocellular carcinoma patients and detectable in human blood [12]. *PCA3* (prostate cancer gene 3) is detectable in urine of prostate cancer patients, showing high accuracy [13]. In addition, *MALAT1* (metastasis-associated lung adenocarcinoma transcript 1) might be a candidate biomarker for NSCLC [14]. *MALAT1* is a well-described lncRNA widely expressed in normal tissues [15]. In several human carcinomas *MALAT1* was shown to be upregulated [16], particularly in early-stage metastasizing NSCLC.

The aim of this study was the evaluation of *MALAT1* as a blood-based biomarker for NSCLC. The expression of *MALAT1* was measured in the cellular fraction of peripheral human blood and the expression levels of NSCLC patients and cancer-free controls of the general population were compared.

## Methods

### Study population

The study was designed according to rules guarding patient privacy and with the approval from the ethics committee of the Ruhr-Universität Bochum (No. 3217–08). All participants provided written informed consent.

The cancer group of 45 NSCLC patients consisted of 21 patients with AdCa (adenocarcinoma) and 24 patients with SqCC (squamous cell carcinoma). Participants were recruited at the HELIOS Clinic Emil von Behring, Berlin, Germany. Tumor staging was performed according to the TNM classification of malignant tumors [17]. Cancer patients had not been treated by surgery, chemotherapy, or radiation therapy before blood collection. The control group of 25 cancer-free subjects was drawn from the Heinz Nixdorf Recall study, a population-based cohort of elderly subjects [18]. Characteristics of the study groups are summarized in Table 1. Detailed subject characteristics are listed in Additional file 1.

**Table 1:** Characteristics of the study groups comprising patients with NSCLC (non-small cell lung cancer), subdivided into AdCa (adenocarcinoma) and SqCC (squamous cell carcinoma), and cancer-free controls

		**Total**	**NSCLC**	**AdCa**	**SqCC**	**Controls**
N		70	45	21	24	25
Gender	Male	48	30	15	15	18
	Female	22	15	6	9	7
Age (years)	Median	69	68	68	67	71
	Range	54 - 84	54 - 83	57 - 83	54 - 81	56 - 84
Smoking status	Ever	64	44	20	24	20
	Never	6	1	1	0	5
Tumor stage	I/II		3	0	3	
	III/IV		42	21	21	

### RNA isolation

Peripheral blood samples were collected from each participant in 9.0 ml S-Monovette EDTA gel tubes (Sarstedt, Nümbrecht, Germany) and centrifuged (2000 x g for 10 minutes) within 30 minutes after collection. The cellular fraction was separated from plasma and stored frozen until RNA isolation.

Samples were thawed at room temperature and RNA isolation including DNase I treatment was performed from 0.5 ml of the cellular fraction using the RiboPure Blood Kit according to the manufacturers’ instructions (Life Technologies, Darmstadt, Germany).

### Quantitative real-time PCR (qRT-PCR)

TaqMan assays (Life Technologies) were used for quantitative expression analyses of *MALAT1* (Hs00273907_s1) as potential biomarker and of *GAPDH* (Hs99999905_m1), *HPRT1* (Hs02800696_m1), and *RPLP0* (Hs99999902_m1) as potential reference genes for normalization. Quantitative real-time PCR (qRT-PCR) was performed using a 7900 HT Fast Real-Time PCR System (Life Technologies). For the reverse transcription reaction 12 μl RNA and for the PCR reaction 5 μl cDNA were used as templates. Samples were analyzed in duplicate and non-template controls were included. For cycle threshold (Ct) estimation a fixed threshold of 0.2 was used. Ct values > 35 were considered to be under the detection limit [19] and marked as 35 for analysis [20]. Raw Ct values are presented in Additional file 1.

The performance of potential references was analyzed utilizing RefFinder [21], a web-based comprehensive tool (http://www.leonxie.com/referencegene.php), including the four commonly used algorithms geNorm [22], NormFinder [23], BestKeeper [24], and comparative ΔCt method [25], to evaluate the most stable reference across study groups. As the geometric mean (GM) of several reference genes is more reasonable than a single reference gene [22], the GM of potential references was calculated. Normalized *MALAT1* levels were expressed as ΔCt, with ΔCt = Ct_(*MALAT1*)_ - Ct_(Reference)_.

### Statistical analysis

Median and inter-quartile range (IQR) were used to describe the distribution of *MALAT1* levels. Groups were compared using the non-parametric Kruskal-Wallis test for continuous variables. Sensitivity and specificity of *MALAT1* were determined from receiver operating characteristic (ROC) curves illustrating the performance of *MALAT1* to discriminate the studied groups. In brief, NSCLC vs. controls, AdCa vs. controls, SqCC vs. controls, and AdCa vs. SqCC were analyzed. The bootstrap procedure (1000 runs) was used for internal validation of the estimates in the ROC analyses.

Potential factors influencing *MALAT1* levels were evaluated using a linear regression model. Estimates were given as β with 95% confidence intervals (CI) and p values. Here, values of β > 0 indicate a negative association between the influencing factor and *MALAT1* levels, values of β < 0 a positive association. Logistic regression modeling was performed to estimate the odds ratio (OR) with 95% CI for detecting NSCLC as a function of normalized *MALAT1* levels*.*

Statistical analyses were performed using SAS/STAT and SAS/IML software, version 9.3 (SAS Institute Inc., Cary, NC).

## Results

### Expression stability of candidate references

**Figure 1:**
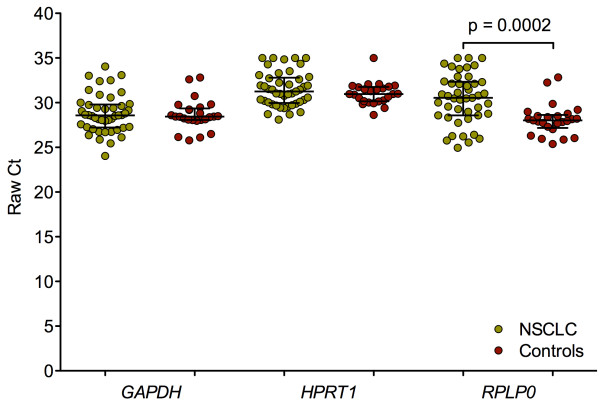
**Scatter dot plots of raw Ct values of candidate reference genes.***GAPDH*, *HPRT1*, and *RPLP0* were measured in patients with NSCLC (non-small cell lung cancer; N = 45) and cancer-free controls (N = 25). Horizontal bars represent median and IQR. Groups were compared using the Kruskal-Wallis test.

The potential reference genes *GAPDH*, *HPRT1*, and *RPLP0* were measured in all samples from NSCLC patients and cancer-free controls. Using raw Ct values no significant differences between NSCLC patients and controls could be observed for *GAPDH* and *HPRT1* in contrast to *RPLP0* (p = 0.0002), (Figure 1). Thus, *RPLP0* was excluded from further evaluation as reference gene.

In order to identify the most stable reference across the study groups RefFinder was used to rank the analyzed references. Lowest rank represents the most stable reference and highest rank represents the least stable reference (Table 2). The GM of *GAPDH* and *HPRT1* was identified as the most stable reference and used for normalization of *MALAT1*.

**Table 2:** **Results of reference analysis using RefFinder [**[21]**] to evaluate the most stable reference across the study groups**

**Algorithm**	**Reference ranking**		
	**1 (most stable)**	**2**	**3 (least stable)**
RefFinder (comprehensive ranking)	GM*	*GAPDH*	*HPRT1*
geNorm	GM* &*GAPDH*		*HPRT1*
NormFinder	GM*	*GAPDH*	*HPRT1*
BestKeeper	*HPRT1*	GM*	*GAPDH*
Comparative ΔCt method	GM*	*GAPDH*	*HPRT1*

*Geometric mean (GM) was calculated from *GAPDH* and *HPRT1.*

### Distribution of MALAT1 in the study groups

Table 3 depicts the distribution of normalized *MALAT1* levels in the study groups. The median normalized *MALAT1* value for NSCLC was -0.35 (IQR -1.34; 1.00), for AdCa -0.59 (IQR -1.34; -0.25), and for SqCC -0.23 (IQR -1.31; 1.47), whereas for controls the median normalized *MALAT1* level was -2.07 (IQR -2.53; -0.83).

**Table 3:** **Distribution of normalized** ***MALAT1*** **levels by median and inter-quartile range (IQR) stratified by potential influencing factors**

		**Total**			**Controls**			**NSCLC**			**AdCa**			**SqCC**		
		**N**	**Median**	**IQR**	**N**	**Median**	**IQR**	**N**	**Median**	**IQR**	**N**	**Median**	**IQR**	**N**	**Median**	**IQR**
Total		70	-0.86	-2.10; -0.05	25	-2.07	-2.53; -0.83	45	-0.35	-1.34; 1.00	21	-0.59	-1.34; 0.25	24	-0.23	-1.31; 1.47
Gender	Male	48	-0.80	-2.18; -0.14	18	-2.18	-2.54; -0.64	30	-0.35	-1.30; 0.97	15	-0.35	-1.44; 0.83	15	-0.36	-1.25; 1.32
	Female	22	-1.01	-2.10; -0.26	7	-1.49	-2.24; -0.92	15	-0.35	-1.37; 1.54	6	-0.84	-1.34; -0.35	9	0.26	-1.37; 1.97
Age	< 60 years	11	-1.93	-2.69; 0.25	4	-2.77	-3.58; -1.49	7	-1.10	-2.01; 0.26	2	-1.14	-2.54; 0.25	5	-1.10	-1.93; 0.26
	60-69 years	26	-0.78	-1.69; -0.05	7	-1.69	-2.24; -0.83	19	-0.41	-1.35; 1.00	9	-0.89	-1.34; -0.05	10	-0.19	-0.74; 1.47
	70-79 years	27	-0.78	-2.29; -0.83	11	-2.07	-2.53; -0.61	16	-0.29	-1.37; 2.27	8	-0.57	-1.37; 1.72	8	0.54	-1.39; 2.64
	≥ 80 years	6	-0.78	-1.25; -0.35	3	-0.92	-2.64; -0.64	3	-0.35	-1.25; 0.24	2	-0.06	-0.35; 0.24	1	-1.25	-1.25; -1.25
Smoking status	Ever	64	-0.86	-2.12; 0.10	20	-2.15	-2.53; -0.87	44	-0.36	-1.35; 0.99	20	-0.69	-1.39; 0.25	24	-0.23	-1.31; 1.47
	Never	6	-1.01	-2.10; -0.61	5	-1.38	-2.10; -0.64	1	1.54		1	1.54				
Tumor size	I/II							3	-1.10	-2.17; -0.41				3	-1.10	-2.17; -0.41
	III/IV							42	-0.29	-1.34; 1.32	21	-0.59	-1.34; 0.25	21	-0.15	-1.25; 1.47
Metastasis status	M0							7	-0.41	-1.93; 1.47	2	-0.22	-1.44; 1.00	15	-0.41	-2.01; 1.91
	M1							19	-0.29	-1.17; 0.54	9	-0.59	-1.34; 0.25	9	-0.15	-0.24; 1.32
Lymph node status	N0							3	0.24	-2.17; 1.32	1	0.24		2	-0.42	-2.17; 1.32
	N1-N3							42	-0.36	-1.34; 1.00	20	-0.69	1.39; 0.54	22	-0.23	-1.25; 1.47

**Figure 2:**
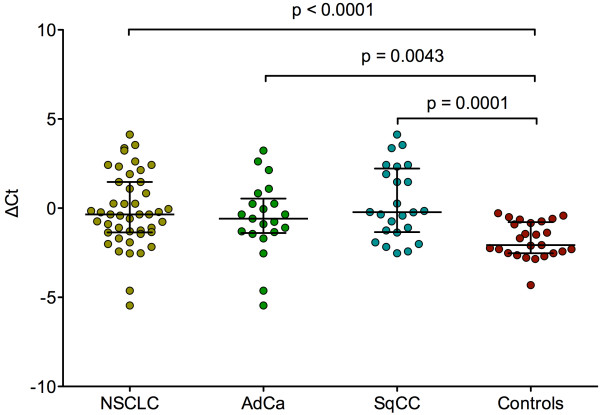
**Scatter dot plots of *MALAT1 *levels in NSCLC (N = 45), AdCa (N = 21), SqCC (N = 24), and cancer-free controls (N = 25).** Expression values were normalized by the geometric mean of *GAPDH* and *HPRT1* and expressed as ΔCt. Horizontal bars represent median and IQR. Groups were compared using the Kruskal-Wallis test. NSCLC: non-small cell lung cancer, AdCa: adenocarcinoma, SqCC: squamous cell carcinoma.

Differences of normalized *MALAT1* levels between cancer patients and cancer-free subjects were significant for NSCLC vs. controls (p < 0.0001), AdCa vs. controls (p = 0.0043), and SqCC vs. controls (p = 0.0001), whereas the difference between AdCa and SqCC was not significant (Figure 2).

### MALAT1 as biomarker of NSCLC

**Figure 3:**
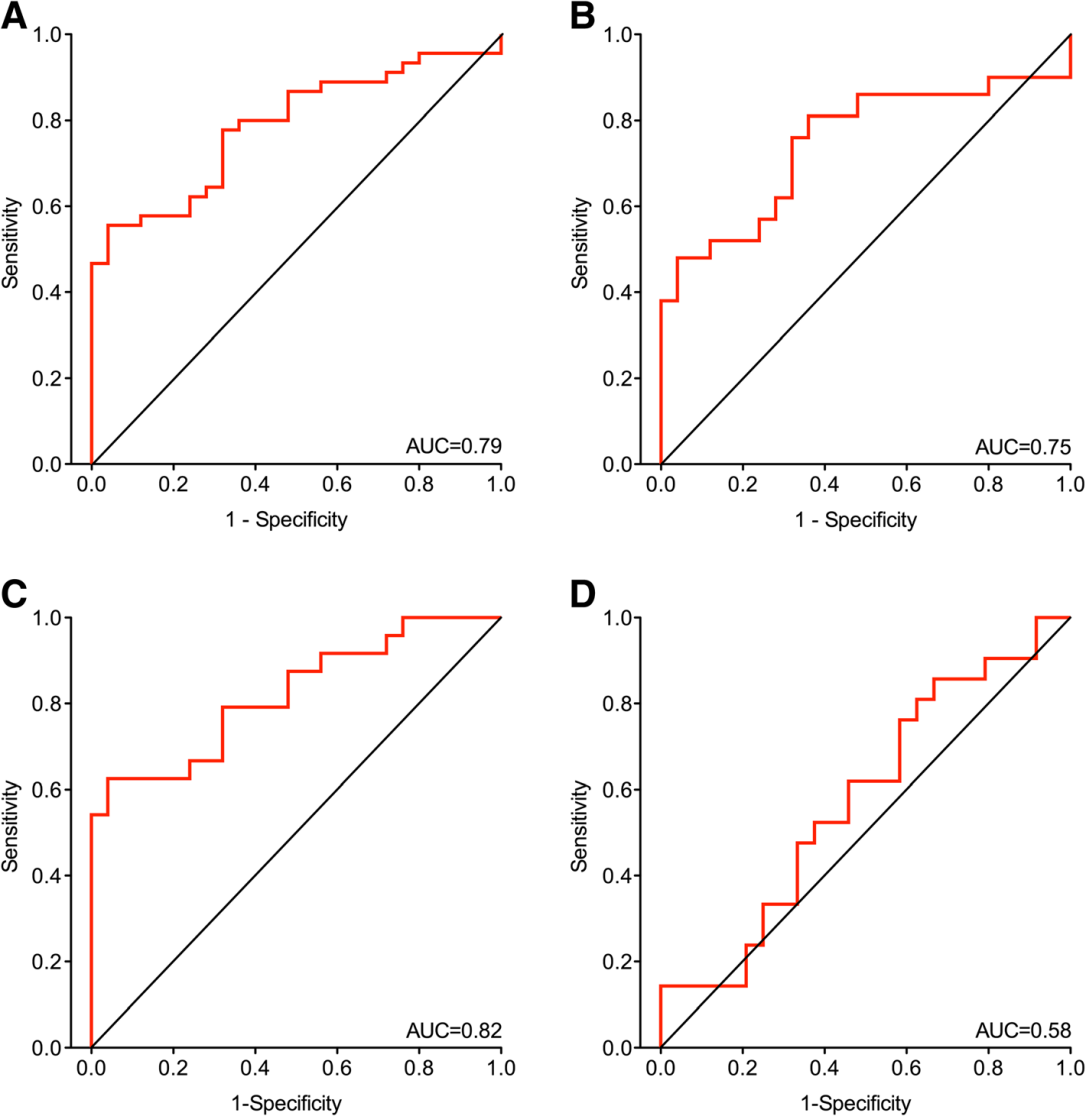
**Receiver operating characteristics (ROC) curves of normalized *MALAT1*.** The area under curve (AUC) was determined for *MALAT1* of **(A)** NSCLC (non-small cell lung cancer) patients and controls, **(B)** AdCa (adenocarcinoma) patients and controls, **(C)** SqCC (squamous cell carcinoma) patients and controls, and **(D)** AdCa patients and SqCC patients.

Using ROC analyses, for NSCLC patients and controls an area under the curve (AUC) of 0.79 (95% CI 0.68 – 0.89), (Figure 3A), for AdCa patients and controls an AUC of 0.75 (95% CI 0.59 – 0.90), (Figure 3B), for SqCC patients and controls an AUC of 0.82 (0.70 – 0.94), (Figure 3C), and for AdCa vs. SqCC an AUC of 0.58 (95% CI 0.42 – 0.76), (Figure 3D), were calculated.

Sensitivity and specificity of normalized *MALAT1* are shown in Table 4, calculated due false-positive rates (FPR) of 0% (no false-positive test), 4% (one false-positive test), and 8% (two false-positive tests), and to the maximum Youden’s Index (YI = sensitivity + specificity -1), respectively. A FPR of 4%, representing 96% specificity, resulted in 56% sensitivity for the discrimination of NSCLC from controls. The sensitivity to discriminate SqCC from controls is higher (63%) than the sensitivity to discriminate AdCa from controls (48%). A FPR of 8% (92% specificity) did not lead to any increase in sensitivities, whereas a FPR of 0% (100% specificity) resulted in lowest sensitivities for the discrimination of controls from patients with NSCLC (47%), AdCa (38%), or SqCC (54%). Use of the maximum YI leads to an increase in sensitivity to 81% only for AdCa vs. controls, but specificity decreased to 64%. For the discrimination of AdCa from SqCC a FPR of 5% (95% specificity, one false-positive test) resulted in 8% sensitivity and a FPR of 10% (90% specificity, two false-positive tests) resulted in 21% sensitivity, whereas using the maximum YI resulted in 33% sensitivity and 86% specificity.

**Table 4:** **Sensitivity and specificity of normalized** ***MALAT1*** **and number of true-positive, true-negative, false-positive, and false-negative tests, calculated for different false positive rates (FPR), i.e., none, one, and two false-positive tests and maximum Youden’s Index (YI)**

		**Cutoff**	**Sensitivity (%)**	**Specificity (%)**	**True-positive (N)**	**True-negative (N)**	**False-positive (N)**	**False-negative (N)**
NSCLC vs. controls	FPR = 0%	-0.24	47	100	21	25	0	24
	FPR = 4%	-0.41	56	96	25	24	1	20
	FPR = 8%	-0.42	56	92	25	23	2	20
	Maximum YI	-0.41	56	96	25	24	1	20
AdCa vs. controls	FPR = 0%	-0.05	38	100	8	25	0	13
	FPR = 4%	-0.35	48	96	10	24	1	11
	FPR = 8%	-0.42	48	92	10	23	2	11
	Maximum YI	-1.44	81	64	17	16	9	4
SqCC vs. controls	FPR = 0%	-0.24	54	100	13	25	0	11
	FPR = 4%	-0.41	63	96	15	24	1	9
	FPR = 8%	-0.42	63	92	15	23	2	9
	Maximum YI	-0.41	63	96	15	24	1	9
AdCa vs. SqCC	FPR = 0%	3.37	8	100	2	21	0	22
	FPR = 5%	2.76	8	95	2	20	1	22
	FPR = 10%	1.91	21	90	5	19	2	19
	Maximum YI	1.32	33	86	8	18	3	16

ROC analyses on 1000 bootstrap samples resulted in similar cutoffs, sensitivities, and specificities of *MALAT1* in comparison to the original analyses. The calculated 95% CI regarding NSCLC vs. controls and SqCC vs. controls indicate a good precision of this assessment, whereas AdCa vs. controls the 95% CI shows a less precision (Additional file 2).

The application of logistic regression models revealed a two-fold increased risk of detecting NSCLC per normalized *MALAT1* unit. The OR of *MALAT1* was 1.88 (95% CI 1.26 - 2.83) without adjustment and 2.03 (95% CI 1.30-3.16) with adjustment for gender, age and smoking status.

### Potential factors influencing MALAT1

The influence of tumor characteristics on *MALAT1* levels is shown in Table 5. *MALAT1* is not affected by tumor size, metastasis status or lymph node status.

**Table 5:** **Analysis of tumor characteristics influencing** ***MALAT1*** **levels in human blood**

		**NSCLC**			**AdCa**			**SqCC**		
		**β**	**95% CI**	**p value**	**β**	**95% CI**	**p value**	**β**	**95% CI**	**p value**
Intercept		-1.23	-3.49; 1.03					-1.23	-3.41; 0.96	
Tumor size (Reference: I/II)	III/IV	1.06	-1.28; 3.39	0.3670				1.52	-0.82; 3.85	0.1911
Intercept		-0.15	-1.11; 0.80		-0.22	-3.24; 2.81		-0.14	1.15; 0.86	
Metastasis status (Reference: M0)	M1	-0.14	-1.36; 1.07	0.8143	-0.46	-3.64; 2.72	0.7655	0.65	-0.98; 2.26	0.4158
Intercept		-0.20	-2.48; 2.08		0.24	-4.02; 4.51		-0.42	-3.20; 2.35	
Lymph node status (Reference: N0)	N1-N3	-0.04	-2.40; 2.32	0.9714	-0.92	-5.29; 3.45	0.6654	0.57	-2.32; 3.47	0.6860

Values of β > 0 indicate a negative association between the analyzed factor and *MALAT1* levels, values < 0 a positive association.

The impact of potential influencing factors on the expression levels of *MALAT1* are shown in Table 6. NSCLC showed a significant 1.63-fold (95% CI 0.75 – 2.51) decrease of *MALAT1* (p = 0.0003), whereas the factors gender, age, and smoking status showed no impact on the *MALAT1* levels in human blood.

**Table 6:** **Analysis of potential factors influencing** ***MALAT1*** **levels in human blood**

		**β**	**95% CI**	**p value**
Intercept		-4.64	-8.93; -0.35	
Group (Reference: controls)	NSCLC	**1.63**	**0.75; 2.51**	**0.0004**
Gender (Reference: male)	Female	0.22	-0.66; 1.10	0.6210
Age	[10 years]	0.45	-0.10; 1.00	0.1058
Smoking status (Reference: never)	Ever	-0.42	-1.94; 1.10	0.5848

Statistically significant changes are marked in bold.

Values of β > 0 indicate a negative association between analyzed factor and *MALAT1*, values < 0 a positive association.

## Discussion

NSCLC is commonly detected in late stages of the disease. Biomarkers have the potential to detect cancer at early stages, facilitating an earlier and therefore more curative therapy that ideally results in decreased mortality. In NSCLC, Gutschner et al. showed that *MALAT1* regulates the expression of several metastasis-associated genes, e.g. *CDCP1* (CUB domain containing protein 1) and *GPC6* (glypican 6), indicating a major role of *MALAT1* in disease progression [26]. Additionally, it was suggested that *MALAT1* might also regulate other important cellular processes in lung cancer [26]. Thus, *MALAT1* is a candidate biomarker for NSCLC [14].

For quantitative expression analysis of messengerRNAs (mRNAs) and miRNAs qRT-PCR is considered to be the gold standard [27] and the same might be true for lncRNAs. However, to produce reliable data in qRT-PCR assays the use of appropriate reference genes for normalization is an important issue [28] and candidate reference genes need to be tested prior to application [29]. As no information regarding lncRNAs as references were accessible, mRNAs were selected as potential references. *HPRT1* and *RPLP0* are well-described reference genes for analyses in NSCLC tissues [30] and *GAPDH* was already applied for normalization of *MALAT1*[15]. However, in this study *RPLP0* seems to be no feasible reference which is in agreement with Falkenberg et al., showing that *RPLP0* is not appropriate as reference gene in human blood samples [31]. In this study, *GAPDH* and *HPRT1* were suitable reference genes, particularly the GM of *GAPDH* and *HPRT1* showed the best reference performance. This is in accordance with Ulivi et al., using *GAPDH* and *HPRT1* for normalization of mRNAs in blood samples of NSCLC patients and controls [32].

One key characteristic of proper diagnostic biomarkers is the need to be detectable in easily accessible body fluids like peripheral blood. In this study *MALAT1* was measured in the cellular fraction of human blood, showing that this matrix is in principle appropriate for the analysis of lncRNAs. Comparable results for the usability of the cellular blood fraction were shown for miRNAs [33,34]. Commonly, the cellular fraction obtained during plasma preparation is discarded, but it might be reasonable to collect this matrix in biobanks for subsequent biomarker discovery.

In this study, a significant downregulation of *MALAT1* in NSCLC patients in comparison to cancer-free controls was shown. Comparable results were achieved by Zhang et al., showing a downregulation of *MALAT1* in patients with hepatocellular carcinomas [35]. However, *MALAT1* was implicated to play an oncogenic role [10] and upregulation of *MALAT1* was observed in several other cancers, e.g. of the breast and prostate [16]. Such differences might be caused by the paradigm that in fact *MALAT1* is expressed ubiquitously but fulfills tissue-specific functions depending on the cellular environment [36]. Commonly, *MALAT1* is analyzed in tissues [14-16], whereas in this study and the study of Zhang et al. [35]*MALAT1* was detected in blood. Because *MALAT1* was detected in the cellular fraction of blood, it is unlikely to be directly produced by the tumor tissue. Its downregulation in blood cells may be an indirect effect of the tumor, e.g., on the immune system. The source of *MALAT1* in the cellular fraction of blood remains unclear. Theoretically, it might originate from leucocytes altered by the tumor. However, further analyses are needed to evaluate the origin of *MALAT1* in human blood. Very recently, it was shown that *MALAT1* was detectable in plasma of patients with gastric or prostate cancer [37,38]. Thus, it would be reasonable to analyze *MALAT1* in plasma of NSCLC patients instead of the cellular fraction, because the presence of *MALAT1* in plasma might be a direct effect of the tumor, e.g., release of lncRNA-containing extracellular vesicles [39].

Tani et al. showed that the stability of *MALAT1* varied in various cell types and indicated that the half-life of *MALAT1* is shorter than the median half-life of mRNA [40]. Such decay of *MALAT1* might also prevail in blood cells. It is well known that systems like PAXgene, Tempus, and RNAlater stabilize mRNAs and miRNAs in whole blood samples [41-43]. Thus, the performance of the assay was additionally tested in a few available blood samples stabilized by PAXgene or RNAlater. In the stabilized blood samples *MALAT1* is detectable at lower Ct values corresponding to larger quantities (data not shown). The results implicate that the use of stabilization systems might be meaningful for lncRNA analyses in blood. However, this assumption needs to be verified in more detail.

Regarding the key characteristics of an obligatory high specificity and a sufficiently high sensitivity of diagnostic biomarkers, the candidate biomarker *MALAT1* does not fulfill both criteria. Generally, in screening cohorts a high specificity is needed to avoid an unacceptably high number of false-positive tests that would result in psychological pressure and needless intervention for the patients. Thus, the sensitivity of candidate biomarkers should be calculated at a fixed high specificity level [44]. In regard to the relatively small study group the specificity of 96% is quite high, particularly as this corresponds to only one single false-positively tested control. On the other hand, the calculated sensitivity is too low (56%) for the use of *MALAT1* as a single biomarker for the diagnosis of NSCLC, particularly for the subtype AdCa (48%). However, lower sensitivity could be balanced by the use of several biomarkers in a panel. Theoretically, in an optimal panel every single biomarker is characterized by sufficiently high sensitivity and the necessary high specificity, perfectly complement each other in order to obtain superior diagnostic performance [45]. Thus, it might be reasonable to verify *MALAT1* in combination with other biomarkers in larger study groups to improve the entire diagnostic performance of the biomarker panel. However, for the discrimination of AdCa and SqCC, a sensitivity of only 8% precludes *MALAT1* as a biomarker for the differential diagnosis of NSCLC subtypes.

Bootstrap analysis showed that the calculated cutoffs, sensitivities, and specificities remain stable, indicating that the calculated values are appropriate for the discrimination of patients and controls.

Regarding the fourth key characteristic of diagnostic biomarkers, the results indicate that *MALAT1* values in blood are not correlated with tumor size, metastasis status, or lymph node status. However, more cases of early-stage metastasizing NSCLC need to be analyzed in subsequent studies because this study comprises only three cases with tumor stage T1 or T2. Additionally, *MALAT1* seems to be relatively independent from common influencing factors like age, gender, and smoking status, indicating the robustness of the candidate biomarker. These observations are in agreement with *MALAT1* expression in tissue [46]. However, it has to be clarified if other potential influencing factors from the multitude of biological, preanalytical, and analytical factors show an impact on *MALAT1* levels in human blood.

## Conclusions

*MALAT1* could be detected in peripheral blood, showing different expression levels between NSCLC patients and cancer-free controls. It was shown that *MALAT1* complies with key characteristics of diagnostic biomarkers, being minimally-invasive, exhibiting high specificity, and robustness. On the contrary, the observed sensitivity is too low for the use of *MALAT1* as a single biomarker for the diagnosis of NSCLC using the cellular fraction of peripheral blood. However, it might be reasonable to verify the performance of *MALAT1* as a complementary biomarker within a panel in larger studies including more cases of early-stage metastasizing NSCLC.

## Competing interests

The authors declare that they have no competing interests.

## Authors’ contributions

DGW conceived of the study, participated in its design and coordination, and drafted the manuscript. GJ participated in study design and coordination and helped to draft the manuscript. SC performed the statistical analyses and helped to draft the manuscript. OB performed the experiments and helped to draft the manuscript. BP and KHJ participated in the statistical analysis and helped to draft the manuscript. JK participated in study design and helped to draft the manuscript. TB participated in study coordination and helped to draft the manuscript. All authors read and approved the final manuscript.

## Supplementary Material

Additional file 1 **Subject characteristics and raw data of *MALAT1, GAPDH*, and *HPRT1 *** **expression analysis.** Click here for file

Additional file 2 Marker cutoffs with 95% CI for NSCLC (non-small cell lung cancer) vs. controls, AdCa (adenocarcinoma) vs. controls, SqCC (squamous cell carcinoma) vs. controls, and AdCa vs. SqCC after bootstrap analysis with 1000 random samples, according to false positive rates (FPR) corresponding to none, one, and two false-positive tests and maximum Youden’s Index (YI). Click here for file
